# The Landscape of Novel Expressed Chimeric RNAs in Rheumatoid Arthritis

**DOI:** 10.3390/cells11071092

**Published:** 2022-03-24

**Authors:** Rajesh Detroja, Sumit Mukherjee, Milana Frenkel-Morgenstern

**Affiliations:** Cancer Genomics and BioComputing of Complex Diseases Lab, Azrieli Faculty of Medicine, Bar-Ilan University, Safed 1311502, Israel; rajesh.detroja@biu.ac.il (R.D.); sumit.mukherjee@biu.ac.il (S.M.)

**Keywords:** autoimmunity, chimeric RNA, fusion gene, rheumatoid arthritis

## Abstract

In cancers and other complex diseases, the fusion of two genes can lead to the production of chimeric RNAs, which are associated with disease development. Several recurrent chimeric RNAs are expressed in different cancers and are thus used for clinical cancer diagnosis. Rheumatoid arthritis (RA) is an immune-mediated joint disorder resulting in synovial inflammation and joint destruction. Despite advances in therapy, many patients do not respond to treatment and present persistent inflammation. Understanding the landscape of chimeric RNA expression in RA patients could provide a better insight into RA pathogenesis, which might provide better treatment strategies and tailored therapies. Accordingly, we analyzed the publicly available RNA-seq data of synovium tissue from 151 RA patients and 28 healthy controls and were able to identify 37 recurrent chimeric RNAs found to be expressed in at least 3 RA samples. Furthermore, the parental genes of these 37 recurrent chimeric RNAs were found to be differentially expressed and enriched in immune-related processes, such as adaptive immune response and the positive regulation of B-cell activation. Interestingly, the appearance of 5 coding and 23 non-coding chimeric RNAs might be associated with regulating their parental gene expression, leading to the generation of dysfunctional immune responses, such as inflammation and bone destruction. Therefore, in this paper, we present the first study to demonstrate the novel chimeric RNAs that are highly expressed and functional in RA.

## 1. Introduction

Chimeric RNAs are produced by the fusion of exons/introns from two different genes [1]. Several recent studies have demonstrated the functional significance of various chimeric RNAs in cancer development and other genetic abnormalities [2,3,4,5,6,7]. Chimeric RNAs could appear in the later stages of cancer, promoting cancer heterogeneity and drug resistance [8,9,10,11]. As such, chimeric RNAs have been recognized as potential biomarkers and drug targets for different cancers [12,13]. Chimeric RNAs could be translated to produce novel fusion proteins, which could alter cell functionality by regulating the dynamics of protein interaction networks [7,14,15]. Chimeric RNAs could also act as long non-coding RNAs (lncRNAs) and play significant regulatory roles that would also help cells to generate new functionalities [16,17,18,19,20,21]. The generation of chimeric RNAs could also increase the functional expansion of cells by creating phenotypic diversity, which would help cells to survive in the face of novel stresses [22]. Therefore, understanding the appearance of chimeric RNAs and their functional association in particular diseases could help us to understand better the complex mechanisms behind disease development.

Rheumatoid arthritis (RA) is an auto-immune disorder exemplified by the chronic and persistent inflammation of the joint synovial tissue associated with the destruction of the affected joints [23]. The pathogenesis of RA is complex and is probably caused by unknown antigens, which are sensitive to specific inherited factors. The recent transcriptomic profiling of the affected synovium and peripheral blood mononuclear cells (PBMCs) from RA patients displayed significant differences in gene expression, which enabled the identification of distinct molecular mechanisms involved in RA pathogenesis [24,25]. RA presents significant clinical heterogeneity [26], as demonstrated by the presence of different auto-antibody specificities, such as rheumatoid factor (RF) and anti-cyclic citrullinated peptide antibodies (ACPA) in the serum [27,28], and different responses to treatment [29,30]. The complexity behind RA pathogenesis, heterogeneity, and clinical response to initial drug therapy is still not well understood [31]. The generation of chimeric RNAs could be crucial towards the development of RA and its clinical heterogeneity. However, no study has to date attempted to investigate the landscape of chimeric RNAs expressed in RA.

To understand the potential impact of chimeric RNAs on RA development and heterogeneity, in this paper, we analyze chimeric RNA expression in publicly available RNA-seq data on synovial fluids from 151 RA patients and 28 healthy controls. We identified 37 recurrent and RA sample-specific chimeric RNAs whose parental genes are predominately involved in immune-related processes, such as “adaptive immune response” and the “positive regulation of B-cell activation”. Furthermore, we found that parental genes of 20 of 37 such chimeric RNAs are significantly differentially expressed in RA samples. This points to these chimeric RNAs being associated with the dysfunctional immune response generation that leads to inflammation and bone destruction. Significantly, our study provides the signature of chimeric RNA expression in RA and highlights the potential clinical importance of this signature in personalized treatments.

## 2. Materials and Methods

### 2.1. Collection of RNA-Seq Data

A total of 217 publicly available total RNA sequencing samples isolated from joint synovial biopsies were downloaded from the GEO database (GEO accession: GSE89408). These contained a total of 151 samples from RA patients, a total of 28 samples from healthy subjects (HT), and a total of 38 samples from other arthritis patients (Table 1). We also downloaded RNA-seq data from tissue samples of 122 healthy human individuals representing 32 different tissues from EBI ArrayExpress (accession E-MTAB-2836) [32] as controls to distinguish RA-specific chimeras. All of the raw sequencing data were initially subjected to quality control analysis using FastQC [33] and an in-house bash script. A total of ~87 million paired-end raw reads of 100 bp were generated for each sample. According to the quality control analysis results, Illumina universal adapter sequences and 11 bp at the start of reads with imbalanced A/T and G/C ratios were trimmed using the cutadapt [34] tool.

### 2.2. Identification of Chimeric RNAs from RNA-Seq Data

All 217 samples were further used to identify chimeric RNAs using our in-house reference-based method ChiTaH [35], previously demonstrated to be the most efficient reference-based tool, as compared to all other available tools used for chimeric RNAs detection. ChiTaH uses 43,466 non-redundant high-quality human chimeras from the ChiTaRS 5.0 database to perform mapping of RNA-Seq datasets and to predict potential chimeric RNAs in each sample.

### 2.3. Differential Gene Expression Analysis

RNA sequencing reads of all 217 samples were subjected to analysis by STAR aligner [36]; a human reference genome (hg38) was used for alignment. Next, the total number of reads mapped to each human gene was calculated using the featureCounts [37] tool. Finally, a read count table of samples from healthy subjects (HT) and RA patients were subjected to differential gene expression analysis using DESeq2 [38]. A gene was considered to be significantly up-regulated when log2foldchange ≥ 2 and Padj ≤ 0.05, whereas a gene was considered to be significantly down-regulated when log2foldchange was negative and Padj ≤ 0.05.

### 2.4. Annotation and Enrichment Analysis of the Parental Genes of Recurrent Chimeric RNAs in RA

Gene ontology, pathway, and gene enrichment analysis of the parental genes of recurrent chimeric RNAs were performed using the online Metascape server [39]. In this analysis, we studied the enrichment of the parental genes of chimeric RNAs in terms of specific biological processes, pathways, and diseases related to autoimmunity.

### 2.5. Classification of Recurrent Chimeric RNAs into Coding and Non-Coding RNAs

The functional classification of recurrent chimeric RNAs was performed based on their protein-coding abilities. Three different tools (CPAT [40], CNIT [41], and LncFinder [42]) were used for this classification. We classified a given chimeric RNA as protein-coding or as a lncRNA when the output of the three tools agreed.

## 3. Results

### 3.1. Identification of Chimeric RNAs across Normal and Arthritis Cohorts

A total of 2102 chimeric RNAs was identified from 151 samples of rheumatoid arthritis patients, as were 856 chimeric RNAs from 22 samples of osteoarthritis patients (Table 2). Moreover, we also found 833 chimeric RNAs in the joint synovial biopsies of 28 healthy individuals, while 2066 chimeric RNAs were identified in 199 samples, representing 122 individuals and 32 different normal tissues. Chimeric RNAs expressed in normal joint synovial biopsies and 32 different normal tissues (EBI samples) were considered as normal or population chimeric RNAs, which could be observed as a result of potential stress response.

### 3.2. Expression Analysis of Recurrent Chimeric RNAs in RA Patients

**Figure 1:**
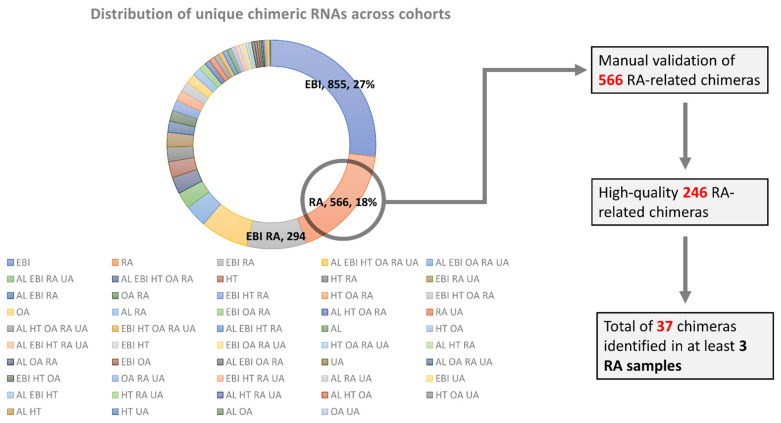
Distribution of the identified chimeric RNAs across cohorts. A total of 566 chimeric RNAs was uniquely identified from 151 RNA-Seq samples of RA patients. After manual validation, a total of 246 RA-specific chimeric RNAs remained. Finally, 37 recurrent chimeric RNAs expressed in at least 3 RA samples were used for further downstream analysis.

We performed a comparative analysis of all identified chimeric RNAs in different cohorts to curate uniquely expressed chimeric RNAs only in 151 RA patients. A total of 566 chimeric RNAs was found to be uniquely expressed in RA patients. Next, according to the manual validation of sequences of 566 chimeric RNAs, a total of 246 high-quality novel chimeric RNAs was obtained (Table S1). Finally, a total of 37 chimeric RNAs was found to be expressed in at least 3 RA patients, which were considered recurrent chimeric RNAs and used for downstream analysis (Figure 1).

### 3.3. Enrichment Analysis of the Parental Genes of RA-Specific Recurrent Chimeric RNAs

**Figure 2:**
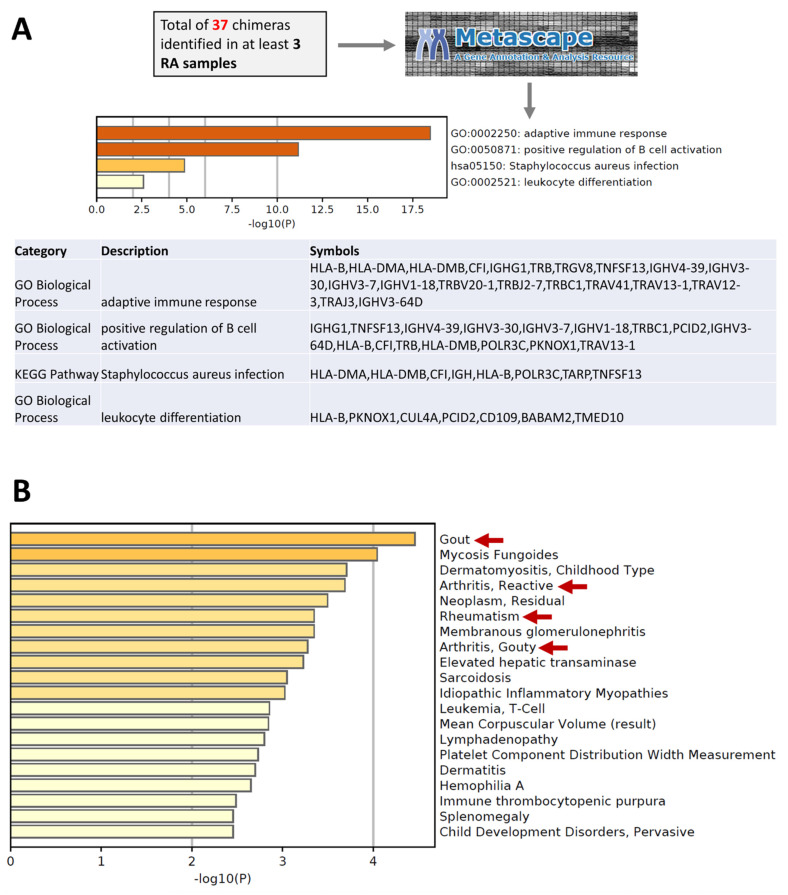
Gene annotation and enrichment analysis of the parental genes of recurrent chimeric RNAs. (**A**) The majority of genes are involved in processes, such as “adaptive immune response” and the “positive regulation of B-cell activation”. Some genes are also involved in “leukocyte differentiation”. (**B**) Disease enrichment analysis shows that these genes are significantly involved in gout arthritis, reactive arthritis, and rheumatism.

Next, a total of 56 unique parental genes of 37 chimeric RNAs was used for gene annotation and enrichment analysis using Metascape [39]. The enrichment of the 56 genes into biological processes showed the majority of genes to be involved in processes, such as “adaptive immune response” and the “positive regulation of B-cell activation” (Table S2). Some of the genes were also involved in “leukocyte differentiation”. Moreover, using Metascape [39], we also studied the enrichment of the 56 genes into different diseases. Disease enrichment analysis shows that these genes were significantly involved in gout arthritis, reactive arthritis, and rheumatism (Figure 2). Altogether, these results suggest that 37 recurrent chimeric RNAs play significant roles in the pathogenicity of rheumatoid arthritis by prompting a dysfunctional immune response in the associated tissues.

### 3.4. Differential Gene Expression Analysis of the Parental Genes of Recurrent Chimeric RNAs

**Figure 3:**
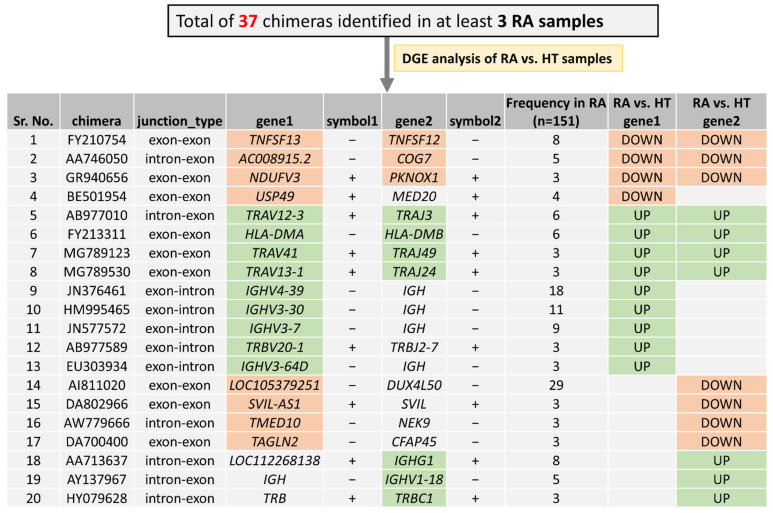
Differentially expressed parental genes of recurrent chimeric RNAs in RA. The parental genes of at least 20 of 37 recurrent chimeric RNAs were differentially expressed. Most of the parental genes were up-regulated (green), while some were down-regulated (orange).

Assessing differential gene expression among samples of joint synovial biopsies from 28 healthy individuals and 151 RA patients was performed using DESeq2 [38]. Such analysis revealed that the 27 parental genes of at least 20 of 37 recurrent chimeric RNAs were significantly differentially expressed (Tables S3–S7). Most of the parental genes were up-regulated, although some were down-regulated (Figure 3). The up-regulated genes are involved in biological processes such as “adaptive immune response” and the “positive regulation of B-cell activation”. Altogether, these results suggest that differentially expressed parental genes of recurrent chimeric RNAs in RA patients could trigger a dysfunctional immune response. Intermediate files for differential gene expression analysis can be retrieved from (https://github.com/Rajesh-Detroja/RA_Chimeric_RNAs/, accessed on 14 February 2022).

### 3.5. Functional Classification of RA-Specific Recurrent Chimeric RNAs

**Figure 4:**
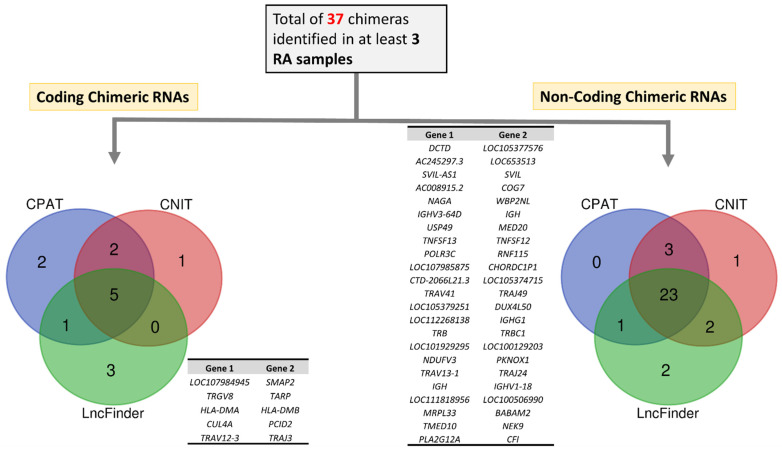
Classification of RA-specific recurrent chimeric RNAs into coding and non-coding gene sequences using CPAT, CNIT, and LncFinder. A total of 5 coding and 23 non-coding chimeric RNAs were identified by all three methods.

To understand further the structure and function of recurrent chimeric RNAs in RA, these were classified as “coding” or “non-coding”. For this analysis, we considered common predictions made using three tools, namely, CPAT [40], CNIT [41], and LncFinder [42]. We thus identified a total of five coding chimeric RNAs with the potential to translate into functional chimeric proteins (Table S8). In contrast, we found a total of 23 non-coding chimeric RNAs with the potential to play similar functional regulatory roles as lncRNAs [Figure 4]. Among these 23 non-coding chimeric RNAs, at least 1 gene of the parental genes of 13 chimeric RNAs is differentially expressed, supporting the potential regulation of parental gene expression. Altogether, these observations suggest that most of the recurrent novel chimeric RNAs in RA patients play a regulatory role as non-coding RNAs, although some can translate into a functional protein. Therefore, the appearance of these novel chimeric proteins or chimeric lncRNAs could be associated with dysfunctional immune response generation, leading to the development of clinical symptoms in RA.

## 4. Discussion

Recent studies identified several chimeric RNAs in different cancers that are associated with oncogenesis, cancer heterogeneity, and the evolution of cancer drug resistance [8,43]. The appearance of chimeric RNAs in a particular cell can promote functional expansion and increase phenotypic diversity to help the cell to survive in the face of new stresses. The process of production of chimeric RNAs has an advantage over point mutations as it involves two parental genes. Therefore, the generation of chimeric RNAs is important for creating the phenotypic plasticity of diseased cells that can provide a fitness advantage that allows a cell to adapt to new disease-related stresses [22]. RA is an auto-immune disorder characterized by synovitis, systemic inflammation, and the presence of auto-antibodies, resulting in progressive joint damage [44]. RA presents disease heterogeneity, leading to treatment failure and remission [45,46]. Therefore, understanding the landscape of chimeric RNA expression in RA could provide new insights into RA development, heterogeneity, and drug response.

**Figure 5:**
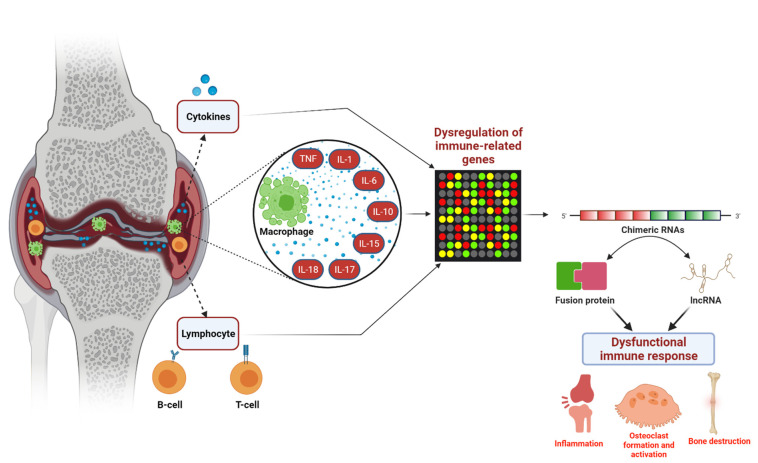
Computational study of expressed and recurrent chimeric RNAs in joint synovial biopsies of RA patients. Genes involved in immune response were significantly dysregulated, which leads to the generation of chimeric RNAs with the potential to translate into function chimeric proteins and regulatory long non-coding RNAs, resulting in the dysfunctional immune responses that cause inflammation and bone destruction.

In the present study, we analyzed publicly available RNA-seq data from disease-relevant and healthy synovial tissues. Our systematic integrative analysis identified 37 recurrent RA-specific chimeric RNAs and several RA sample-specific chimeric RNAs. Previous studies demonstrated recurrent cancer-specific chimeric RNAs to be involved in cancer development. Moreover, chimeric RNAs expressed in specific cancer samples are important for cancer heterogeneity. Therefore, our finding suggests that the expression of recurrent chimeric RNAs in RA patients could be associated with RA development, whereas the expression of RA sample-specific chimeric RNAs could be important for generating disease heterogeneity. Furthermore, to understand whether recurrent chimeric RNAs are associated with RA development, we analyzed the expression of their parental genes and the pathways to which these genes contribute. Interestingly, we observed the parental genes of the 37 recurrent chimeric RNAs to be mainly involved in the immune-related processes and adaptive immune responses, namely, “adaptive immune response” and the “positive regulation of B-cell activation”. We also found that at least one parental gene is differentially expressed in most cases. Therefore, this could indicate that the appearance of chimeric RNAs is associated with the generation of dysfunctional immune responses in RA. Further, we performed the disease enrichment analysis to check the most enriched diseases where these parental genes of RA-specific recurrent chimeric RNAs are involved. We found the most enriched diseases are gout, rheumatism, and reactive arthritis. Therefore, the dysregulation of these genes could induce the common clinical signature of gout and RA, such as pain and swelling and stiffness of joints. Next, the functional characterization of RA-specific recurrent chimeric RNAs predicted the majority of them could act as lncRNA and involved in regulatory functions. In summary, we hypothesize that these recurrent chimeric RNAs could regulate the expression of their parental gene, leading to the generation of dysregulated immune responses in affected tissues, the development of RA, and subsequent inflammation in osteoclast formation and bone destruction of affected tissues (Figure 5).

In summary, our study provides an expression landscape of chimeric RNAs in the affected synovium of RA patients. Our results also highlight the potential functional association of the novel chimeric RNAs in RA development and heterogeneity. The limitations of our study are that it lacks experimental evidence, due to the unavailability of synovium tissue samples from RA patients. Nonetheless, this study opens a new paradigm to explore further chimeric RNA-mediated transcriptional dysregulation leading to RA development and heterogeneity that could help in the design of new treatment strategies.

## Figures and Tables

**Table 1:** Description of the total RNA sequencing samples.

Description	No. of Samples	Average Raw Paired-End Reads	Average Trimmed Paired-End Reads
Rheumatoid Arthritis	151	86,568,972	86,432,629
Healthy	28	84,069,634	83,923,697
Osteoarthritis	22	86,614,846	86,458,277
Arthralgia	10	90,722,882	90,617,905
Undifferentiated Arthritis	6	87,407,948	87,278,313

**Table 2:** Distribution of the identified chimeric RNAs across the normal and arthritis cohorts.

Description	No. of Samples	No. of Chimeric RNAs
Rheumatoid Arthritis	151	2102
Healthy	28	833
Osteoarthritis	22	856
Arthralgia	10	783
Undifferentiated Arthritis	6	671
Healthy Human Tissues From EBI	199	2066

## Data Availability

Intermediate files and resources is available at https://github.com/Rajesh-Detroja/RA_Chimeric_RNAs/, accessed on 20 March 2022.

## Supplementary Materials

The following are available online at https://www.mdpi.com/article/10.3390/cells11071092/s1, Table S1: Expressed chimeric RNAs in rheumatoid arthritis. Table S2: Gene Ontology Analysis of Recurrent chimeric RNAs in rheumatoid arthritis. Table S3: Significantly differentially expressed genes between RA vs. OA. Table S4: Significantly differentially expressed genes between RA vs. HT. Table S5: Significantly differentially expressed genes between OA vs. HT. Table S6: Significantly differentially expressed genes between AL vs. HT. Table S7: Significantly differentially expressed genes between AL vs. OA. Table S8: Classification of RA-specific recurrent chimeric RNAs into coding and non-coding gene.
